# Plastid phylogenomics and plastome evolution in the morning glory family (Convolvulaceae)

**DOI:** 10.3389/fpls.2022.1061174

**Published:** 2022-12-20

**Authors:** Chung-Shien Wu, Chung-I. Chen, Shu-Miaw Chaw

**Affiliations:** ^1^ Biodiversity Research Center, Academia Sinica, Taipei, Taiwan; ^2^ Department of Forestry, National Pingtung University of Science and Technology, Pingtung, Taiwan

**Keywords:** convolvulaceae, plastome, phylogenomics, gene transfer, intron loss

## Abstract

Convolvulaceae, the morning glories or bindweeds, is a large family containing species of economic value, including crops, traditional medicines, ornamentals, and vegetables. However, not only are the phylogenetic relationships within this group still debated at the intertribal and intergeneric levels, but also plastid genome (plastome) complexity within Convolvulaceae is not well surveyed. We gathered 78 plastomes representing 17 genera across nine of the 12 Convolvulaceae tribes. Our plastid phylogenomic trees confirm the monophyly of Convolvulaceae, place the genus *Jacquemontia* within the subfamily Dicranostyloideae, and suggest that the tribe Merremieae is paraphyletic. In contrast, positions of the two genera *Cuscuta* and *Erycibe* are uncertain as the bootstrap support of the branches leading to them is moderate to weak. We show that nucleotide substitution rates are extremely variable among Convolvulaceae taxa and likely responsible for the topological uncertainty. Numerous plastomic rearrangements are detected in Convolvulaceae, including inversions, duplications, contraction and expansion of inverted repeats (IRs), and losses of genes and introns. Moreover, integrated foreign DNA of mitochondrial origin was found in the *Jacquemontia* plastome, adding a rare example of gene transfer from mitochondria to plastids in angiosperms. In the IR of *Dichondra*, we discovered an extra copy of *rpl16* containing a direct repeat of ca. 200 bp long. This repeat was experimentally demonstrated to trigger effective homologous recombination, resulting in the coexistence of intron-containing and -lacking *rpl16* duplicates. Therefore, we propose a hypothetical model to interpret intron loss accompanied by invasion of direct repeats at appropriate positions. Our model complements the intron loss model driven by retroprocessing when genes have lost introns but contain abundant RNA editing sites adjacent to former splicing sites.

## Introduction

Convolvulaceae, commonly known as the morning glories or bindweeds, is a large family sister to Solanaceae (Stefanović et al., 2002) in the order Solanales (AGP IV, 2016). Morning glories comprise approximately 2,000 species in 60 genera (Simões et al., 2022), with a diverse range of morphological characteristics from herbs, shrubs, vines, to parasites *Cuscuta* (dodders). They mostly inhabit tropical areas and include many economically valuable crop (e.g., sweet potato), traditional medicine (e.g., dodders), ornamental (e.g., morning glory), and vegetable (e.g., water spinach) species. Using a few plastid and nuclear markers, early molecular phylogenetic studies classified 12 tribes in Convolvulaceae (Stefanović et al., 2002; Stefanovi´c et al., 2003), but controversies remain at the intertribal and intergeneric levels even when organellar (Lin et al., 2022) and nuclear (Simões et al., 2022) genomic data were used for comparative phylogenetic analyses.

Plastids are cellular organelles and have their own genomes, called plastomes. In general, seed plant plastomes are structurally conserved and contain two inverted repeats (IRs) separating the sequences into a large single copy (LSC) region and a small single copy (SSC) one. Integration of foreign DNA into plastomes was initially thought impossible due to the absence of active DNA import systems in plastids (Smith, 2011). However, plastid mitochondrial (mt) DNA-derived sequences (PTMTs) were later discovered in a handful of species (e.g., Iorizzo et al., 2012a; Iorizzo et al., 2012b; Straub et al., 2013; Ma et al., 2015; Rabah et al., 2017; Raman et al., 2019; Raman et al., 2021), suggesting that acquisition of foreign DNA that shapes plastome complexity is possible, although the mechanism is unclear. Plastomes also undergo rearrangements, including inversions and changes of gene content through deletions and duplications (see review in Wicke et al., 2011). Gene content changes due to IR contraction and expansion frequently occurred during plant evolution (Wang et al., 2008; Zhu et al., 2016). A comparison of plastid transcriptomes has shed light on the effects of inversions on gene expression in conifer plastids (Wu et al., 2021). However, to date there is little conclusive evidence as to the consequences of plastid gene content changes.

Plastids contain several group II introns. Unlike their eubacterial counterparts/ancestors, modern plastid group II introns have lost their mobility and rely on host-encoded factors for splicing (Lambowitz and Zimmerly, 2011). Group II introns are generally 400−800 bp long and possess six major domains (I−VI domains) that form a conserved tertiary structure and an active site for splicing (Kelchner, 2002; Lambowitz and Zimmerly, 2011). Precise deletions of introns have been proposed to be a consequence of retroprocessing operated in plastids (Downie et al., 1991), while localized retroprocessing appears to be a widespread mechanism underlying intron loss in plant mitochondria (Cuenca et al., 2016).

Recent studies of autotrophic plastomes in Convolvulaceae were mainly confined to the genus *Ipomoea* (Eserman et al., 2014; Park et al., 2018; Sun et al., 2019; Laux et al., 2022). However, Lin et al. (2022) compared plastomes across eight Convolvulaceous tribes and found several unusual features, such as atypical IRs, gene and intron losses, and insertions of foreign DNA of unknown origin. These studies suggest that Convolvulaceous plastomes are labile and their complexity might be underestimated. To further explore this variation, we gathered 78 plastomes representing 17 genera from nine of the 12 tribes in the morning-glory family, including *Jacquemontia*, considered to have the greatest sequence divergence among autotrophic Convolvulaceae (Stefanović et al., 2002). These data were used to construct phylogenetic trees, estimate nucleotide substitution rates, and characterize plastomic rearrangements. Furthermore, we report the first PTMT case in Convolvulaceae and propose a repeat-mediated model to interpret intron loss without retroprocessing.

## Materials and methods

### Taxon sampling, DNA and RNA extraction, and sequencing

We collected 19 Convolvulaceae taxa. Their voucher information is shown in **Table S1**. Total DNA was extracted from fresh leaves using the CTAB method described in Stewart and Via (1993). The extracted DNA was used for library construction using Celero™ DNA-Seq Library Preparation Kits. Total RNA was extracted from *D. micrantha* leaves using Plant Total RNA Purification Kits (GeneMark, Taiwan). After DNase I treatment, the NuQuant^®^ Universal Plus mRNA-Seq Kit, designed for poly (A) selection, was used to prepare cDNA libraries. We used the Illumina HiSeq 4000 platform at Genomics BioSci & Tech (New Taipei City, Taiwan) to obtain approximately 10 Gb of paired-end (PE) reads per library.

### Genome assembly and annotation

Adaptors and low-quality reads were trimmed using Trimmomatic v0.36 (Bolger et al., 2014) with default parameters. Plastomes were assembled using NOVOPlasty v4.3 (Dierckxsens et al., 2017) and GetOrganelle v1.7.6.1 (Jin et al., 2020) with our own databases containing available *Ipomoea* plastomes. We merged the results generated by the two assemblers and did base-scale corrections using Pilon v1.24 (Walker et al., 2014). The assembled scaffolds were annotated in Geneious Prime (https://www.geneious.com/ ), with the *I. aquatica* plastome (NC056300) as the reference. Protein-coding genes and tRNAs were further confirmed by aligning them to their orthologs and tRNAscan-SE v2.0 (Chan et al., 2021), respectively.

### Sequence alignments and tree construction

Sequences of 78 plastid protein-coding genes were retrieved from our assemblies and other publicly available plastomes (**Table S2**). Sequence alignments were conducted using MUSCLE (Edgar, 2004) implemented in MEGA 7 (Kumar et al., 2016). Concatenations of the alignments were performed in Geneious Prime, yielding a supermatrix of 77,232 bp for downstream analyses. We used PartitionFinder v2.1.1 (Lanfear et al., 2017) to search the best scheme of data partitions under the Bayesian information criterion (BIC). This scheme was subsequently incorporated in building ML and BI trees using IQ-tree v2.2.0 (Minh et al., 2020) and MrBayes v3.2.7 (Ronquist et al., 2012), respectively. Supporting values for nodes of the ML tree were estimated from 5,000 ultrafast bootstraps. Two independent runs were conducted for 2 × 10^7^ generations and one tree per 1,000 generations was sampled for BI tree construction. The initial 25% of the sampled trees were discarded as burin-in and Tracer v1.7.1 (Rambaut et al., 2018) was used to check if the effective sample size (ESS) exceeded 200 in all parameters.

### Read mapping

DNA and RNA mapping analyses were performed in Geneious Prime, with the option of “Map to Reference” and mappers = “Geneious” for DNA-seq and “Geneious RNA” for RNA-seq reads. Gaps were not allowed during mapping.

### Calculation of absolute synonymous (*RS*) and nonsynonymous (*RN*) substitution rates

To facilitate calculations, the supermatrix mentioned above was trimmed to contain only 68 accessions of unique species. This trimmed matrix was then used to estimate synonymous (*dS*) and nonsynonymous (*dN*) trees using Codeml of the Paml 4.9j package (Yang, 2007) under a branch model and codon frequency = F3 × 4. Molecular dating was conducted using the Paml MCMCTree module. The constrained ages were based on the settings described in Eserman et al. (2014) with a few modifications: the crown group for Solanaceae (23.0–33.9), for Convolvulaceae (47.8–56.0), for Ipomoeeae *s.l.* + *Merremia* (41.2−47.8 MYA). Markov chain Monte Carlo was evaluated for 2 × 10^6^ generations under a HKY85 + G5 model. We set the sampling frequency = 10 and burnin = 25%. Terminal branches leading to species were used to calculate species *RS* and *RN* from dividing *dS* and *dN* branch lengths by the duration of species evolution.

### PCR assays

We verified the existence of mtDNA insertions using PCR and two specific primer sets (Set 1: F1, 5’-ACTTCT GGTTCCGGCGAACG-3’and R1, 5’-GCTAGAATTAGCATTATGAGCTGCTCG-3’; and Set 2: F2, 5’-CAATTCATTCGTAGTAGGATATTGAAACCTC-3’and R2, 5’-AAGCGCATAATTGGTTGAAGATCAC-3’). Targeted fragments were amplified in a 20 μl PCR reaction: 9.6 μl water, 3.2 μl dNTP mixture (2.5 mM each), 1 μl 10 μM of each primer, 0.2 μl TaKaRa LA Taq^®^, 2 μl 10X LA PCR buffer II, 2 μl 25 mM MgCl_2_, and 1 μl of genomic DNA (~25 ng). Amplification conditions were as follows: initial denaturation at 94°C for 3 min., followed by 30 cycles of 98°C for 15 sec., 58°C for 20 sec., and 68°C for 5 min. The PCR was finished with a final step at 72°C for 10.0 min. We also designed PCR primers to verify the coexistence of *rpl16*-LSC (*rpl16*-LSC-F: 5’-GAGAGTTTCTTCTCATCCAGCTCCTC-3’ and *rpl16*-LSC-R: 5’- CGGAACCTGTGAATGCAAAAGATC-3’), *rpl16*-IR (*rpl16*-IR-F: 5’- GATTAGGGTAAACCAGACCCATTCATAGT-3’ and *rpl16*-IR-R: 5’-ATTCTTCCTCTATGTTGTTTACGGAATCTG-3’), and *rpl16*-IR* (*rpl16*-IR*-F: 5’-CTTTTGATATAATTATCATTGCTATGCTTAGTCC-3’ and *rpl16*-IR*-R: 5’-AATTGAGTTCGTATAGGCATTTTGGATG-3’) copies. PCR conditions were similar to those used in examining mtDNA insertions, but the number of PCR cycles was gradually increased from 10 to 30. Electrophoresis was carried out for an hour at 50 V on 0.8% agarose TAE gels and PCR amplicons were visualized in a Quantum CX5 gel image system.

## Results

### Plastid phylogenomics of Convolvulaceae

**Figure 1:**
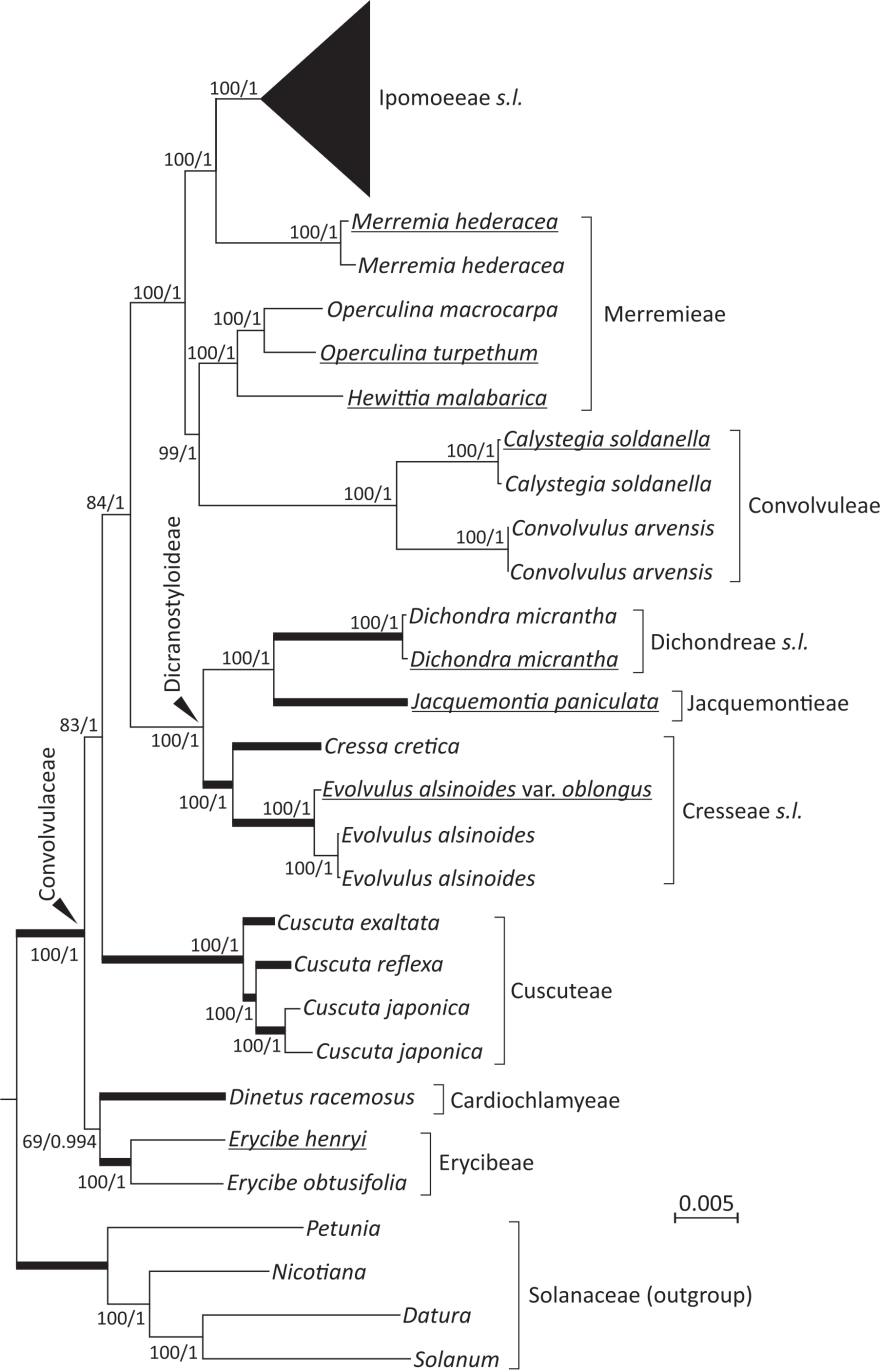
Plastid phylogenomics of representative Convolvulaceous genera and tribes. The topology is based on a ML tree inferred from a concatenation of 78 plastid protein-coding genes. Values along branches reflect bootstrap support (%) for ML trees and posterior possibilities for Bayesian inference (BI) trees. Thick branches are shortened to 1/10 of their real lengths. Underlined taxa were sequenced in this study.

The 19 newly sequenced plastomes are circular molecules. They exhibit typical quadripartite structure of a large single-copy (LSC) region, a small single-copy (SSC) region, and a pair of inverted repeats (IRs). Their sizes range from 152,365 to 165,459 bp and GC content from 37.3 to 38.8%. Using Solanaceae as the outgroup, our ML and BI trees show nearly identical topologies (**Figure 1**; **Figure S1**). Both trees suggest that the tribes Cardiochlamyeae and Erycibeae constitute the earliest diverged clade, followed by Cuscuteae, a solo parasitic tribe in Convolvulaceae. The tribes Cresseae *s.l.*, Dichondreae *s.l.*, and Jacquemontieae form a monophyletic clade sister to the clade comprising the tribes Convolvuleae, Ipomoeeae *s.l.*, and Merremieae. Notably, our trees strongly support (BS = 100% and PP = 1) Merremieae as a paraphyletic tribe because the position of *Merremia* is closer to Ipomoeeae *s.l.* than to other Merremieae genera (**Figure 1**).

Within Ipomoeeae *s.l.*, two separated clades are observed: Argyreiinae and Astripomoeinae, with the former containing the four genera *Argyreia*, *Ipomoea*, *Stictocardia*, and *Turbina* and the latter only *Ipomoea* (**Figure S1**). In the ML tree, the three *I. aquatica* accessions, including two cultivars, are monophyletic and sister to *I. reptans* with weak support (63%). Such relationships, however, are not present in the BI tree under the 50% majority rule (**Figure S1**). To date, *I. reptans* has been considered a synonym for *I. aquatica*. Here, we treat *I. reptans* as a separate species because of its semi-terrestrial habitat, purple stems, and corollaceous color differing from *I. aquatica* (**Figure S1**).

### Extreme variability in plastid nucleotide substitution rates

**Figure 2:**
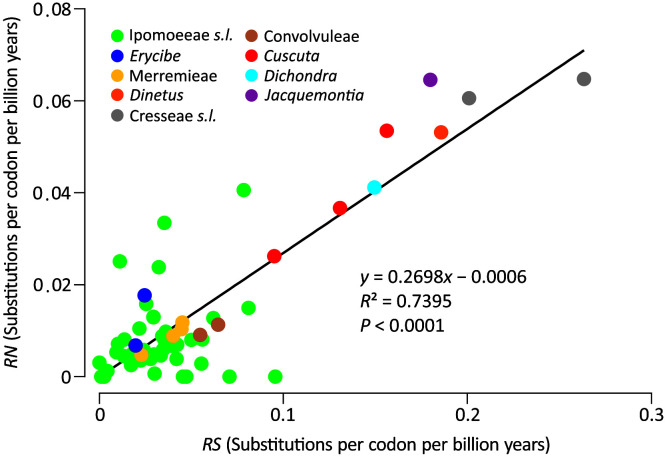
Comparisons of absolute synonymous (*RS*) versus nonsynonymous (*RN*) substitution rates across Convolvulaceae. The solid line is the regression between *RN* and *RS*.

To explicitly assess the variation in substitution rates across Convolvulaceae, synonymous (*dS*) and nonsynonymous (*dN*) trees were inferred from the concatenation of the 78 plastid genes. This concatenated gene set was also used to estimate divergence times among lineages. Our molecular dating suggests that Convolvulaceae split from Solanaceae at ca. 88.7 MYA and divergence of the Convolvulaceous tribes occurred in between 23.5 and 52.8 MYA (**Figure S2**). **Figure 2** depicts absolute synonymous (*RS*) and nonsynonymous (*RN*) substitution rates that allow for rate comparisons on the same time scale. Notably, the *RS* and *RN* are strongly correlated across Convolvulaceae (*R* ^2^ = 0.7395), suggesting their dependent evolution. We noticed that Ipomoeeae *s.l.*, Merremieae, Convolvuleae, and *Erycibe* evolved at an equal rate but significantly slower than *Dichondra*, *Jacquemontia*, Cresseae *s.l.*, *Cuscuta*, and *Dinetus* in *RS* (Mann Whitney test, *P* < 0.05). In particular, the mean *RS* in Cresseae s.l. is approximately seven times faster than in Ipomoeeae s.l. Significantly elevated *RN* is only detected in Cresseae *s.l.* and *Cuscuta* (Mann Whitney test, *P* < 0.05). Taken together, these results show that substitution rates at *dS* sites are highly variable across Convolvulaceae.

### Plastomic rearrangements and IR contraction/expansion

**Figure 3:**
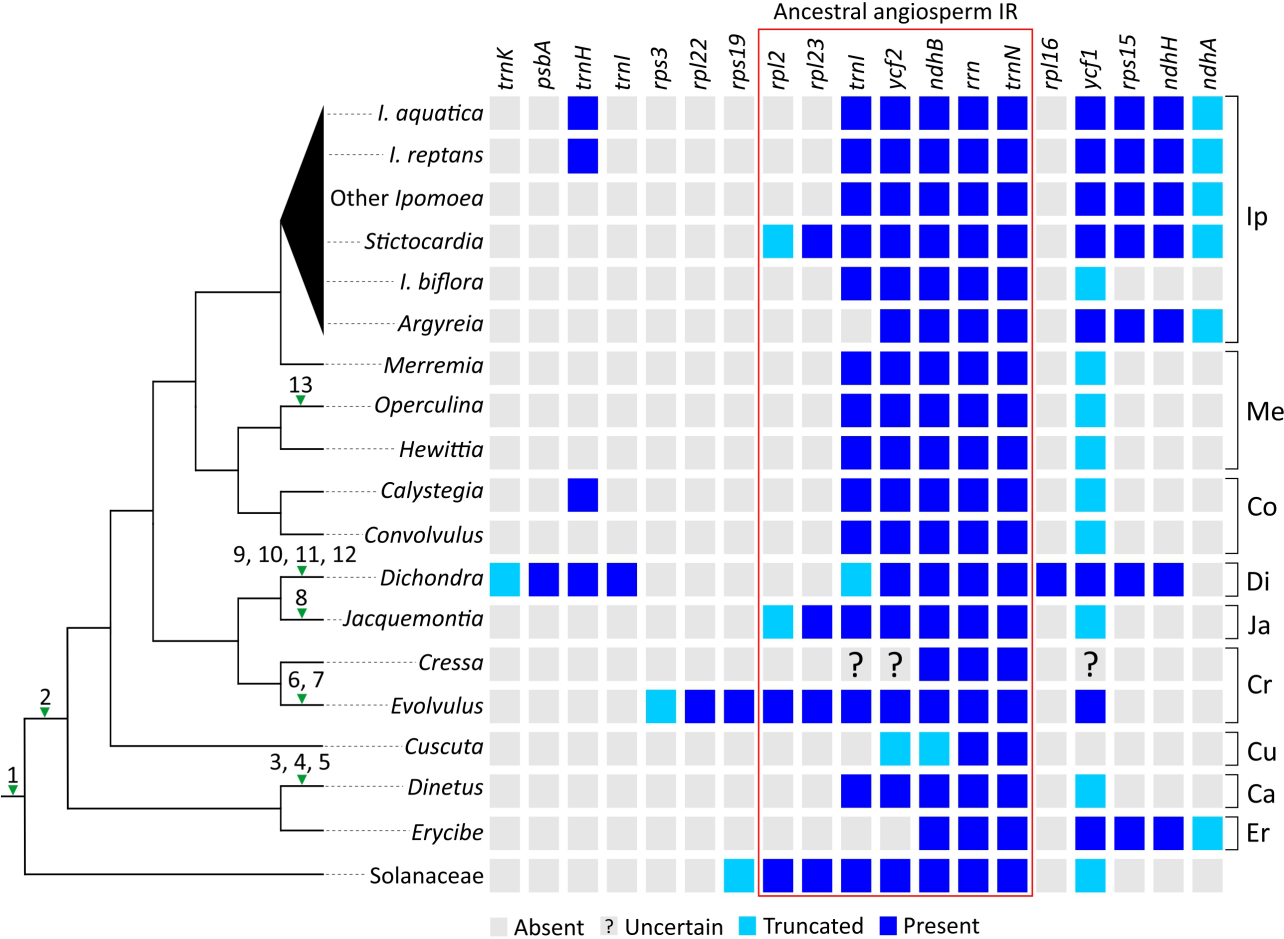
Evolution of plastomic rearrangements across Convolvulaceae. The right panel depicts presences/absences of genes in IRs. A “?” mark indicates that the gene state is uncertain due to poor sequence quality. Numerals on branches of the left panel tree denote specific rearrangements outside IRs. 1, loss of *infA*; 2, loss of the *rpl2* intron; 3, inversion of the *atpI−atpB* region; 4, duplication of *psbT*; 5, loss of *rpl23*; 6, loss of the *rps16* intron; 7, loss of the *rpoC1* intron; 8, integration of PTMTs between *psaA* and *ycf3*; 9, inversion of the *trnL−atpB* region; 10, loss of *rpl23*; 11, loss of the *rps16* intron; 12, loss of all introns from *ycf3*; 13, loss of *rpl23*. Ip, Ipomoeeae *s.l.*; Me, Merremieae; Co, Convolvuleae; Di, Dichondreae *s.l.*; Ja, Jacquemontieae; Cr, Cresseae *s.l.*; Cu, Cuscuteae; Ca, Cardiochlamyeae; Er, Erycibeae.

To sketch plastomic rearrangement profile across Convolvulaceae evolution, we labeled rearrangements in SC regions (hereafter called SC rearrangements) on the tree branches (**Figure 3**; left panel) and compared IR gene content among sampled taxa (**Figure 3**; right panel). SC Rearrangements in *Cuscuta* taxa are not included in the current study because they have been described previously (Braukmann et al., 2013; Banerjee and Stefanović, 2020). We discovered 13 SC rearrangements across the tree. All Convolvulaceous genera, including *Cuscuta*, lack the *rpl2* intron and *infA* gene, and the latter is also absent in Solanaceae (Amiryousefi et al., 2018). This implicates two ancient loss events that occurred before (i.e., *infA*) and after (*rpl2* intron) the split of Convolvulaceae from Solanaceae (**Figure 3**). In contrast, the remaining SC rearrangements are all genus specific. For instance, an inversion of the *atpI−atpB* region, duplication of *psbT*, and loss of *rpl23* in *Dinetus*; integration of mtDNA between *psaA* and *ycf3* in *Jacquemontia*; an inversion of the *trnL−atpB* region, loss of *rpl23*, and losses of *rps16* and *ycf3* introns in *Dichondra*; losses of *rps16* and *rpoC1* introns in *Evolvulus*; and loss of *rpl23* in *Operculina*. Overall, our analyses suggest that *rpl23* has been independently lost at least three times during Convolvulaceae evolution (**Figure 3**).

Using Solanaceae and ancestral angiosperm IRs (Zhu et al., 2016) as the references, we observed that IRs of most Convolvulaceous genera lack *rpl2* and *rpl23* (**Figure 3**). As a result, the most parsimonious scenario is that the common ancestor of Convolvulaceae experienced a contraction so that *rpl2* and *rpl23* were excluded from its IRs, followed by multiple rounds of lineage-specific expansion and contraction that shaped the IR diversity. For example, (1) *Erycibe* and Ipomoeeae *s.l.* have undergone a parallel expansion to include *ycf1*, *rps15*, *ndhH*, and partial *ndhA* in their IRs. This expansion is here characterized as a synapomorphic trait of Ipomoeeae s.l., despite a subsequent contraction removing *rps15*, *ndhH*, and partial *ndhA* from *I. biflora*’s IRs; (2) *Dichondra* has duplicated *psbA*, *trnH*, *trnI*, *rpl16*, *ycf1*, *rps15*, *ndhH*, and partial *trnK* in its IRs, manifesting a complex IR expansion history including genes originally located in the LSC and SSC regions; (3) IR expansions have caused duplications of *rpl23*, *rpl2*, *rps19*, *rpl22*, and partial *rps3* in *Evolvulus* as well as *rpl23* and partial *rpl2* in *Jacquemontia* and *Sticotocardia*, whereas contractions have removed *trnI* and *ycf2* from the *Erycibe* IRs as well as *trnI* from the *Argyreia* IRs; (4) Small IR expansions have led to parallel duplications of *trnH* in *Calystegia*, *I. aquatica*, and *I. reptans*.

### Integration of mtDNA in the *Jacquemontia* plastome

**Figure 4:**
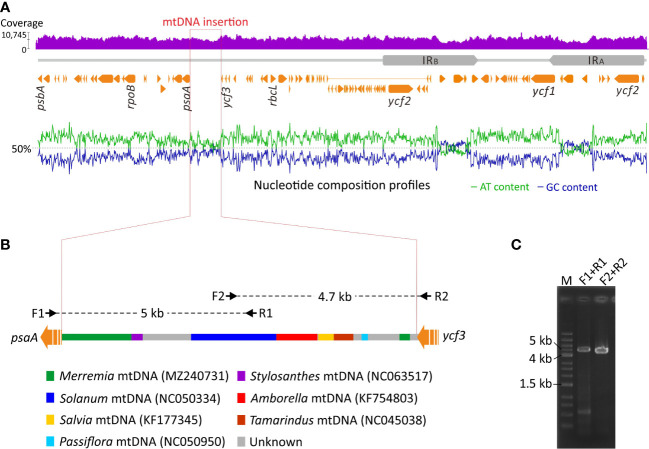
Schematic diagrams of PTMT integration in *Jacquemontia*. **(A)** Analyses of the DNA-seq read coverage and nucleotide composition across the whole plastome. **(B)** Blast results of the integrated PTMT showing a mixture of fragments best matching mtDNAs of some taxa with color codes given below. Black arrows are PCR primers designed in this study. **(C)** PCR verification of the integrated PTMT.

We detected an unusual elongated intergenic spacer between *psaA* and *ycf3* in the *Jacquemontia* plastome. This spacer is 9.1 kb long and approximately nine times longer than those (<1 kb) of other Convolvulaceous taxa. Furthermore, GC content of this intergenic spacer is elevated with cytosines and guanines accounting for 43.1% of all bases, in contrast to an average of 38.6% elsewhere (**Figure 4A**). Using this intergenic spacer sequence of *Jacquemontia* as the query, blast searches (NCBI nr database; access date: Jun/2022) yielded a mixture of fragments best matching mitogenomes of several taxa, including a basal angiosperm (*Amborella*) and other Convolvulaceous genera (**Figure 4B**). Such blast results, however, were not observed in other Convolvulaceous taxa whose syntenic sequences of this intergenic spacer always best matched plastomic sequences. Thus, this intergenic spacer between the *psaA* and *ycf3* genes of *Jacquemontia* could have been mis-assembled with contaminants from endogenous mtDNA, or may be a true integrated PTMT.

To verify the accuracy of our assembly, DNA-seq reads were mapped to the *Jacquemontia* plastome. Given the remarkably different copy numbers of plastomes compared to mitogenomes in plant cells (Johnston, 2019), we would expect drastic decreases of the read coverage in mis-assembled regions. However, our mapping analysis yielded a similar degree of the read coverage across the entire plastome (**Figure 4A**), thus rejecting the assumption that our assembly had endogenous mtDNA contamination. Both NOVOPlasty (Dierckxsens et al., 2017) and GetOrganelle (Jin et al., 2020) assemblers, though based on different assembly strategies, obtained identical results that again confirmed the presence of a PTMT in *Jacquemontia*. We also designed specific primers to amplify the region across the intergenic spacer between *psaA* and *ycf3.* Our PCR successfully yielded fragments of the expected intergenic size (9.1 Kb; **Figure 4C**). Collectively, our data demonstrate an unprecedented case of PTMTs in Convolvulaceae.

### Short, domain-lacking, but repeat-containing introns in duplicated *rpl16* genes

**Figure 5:**
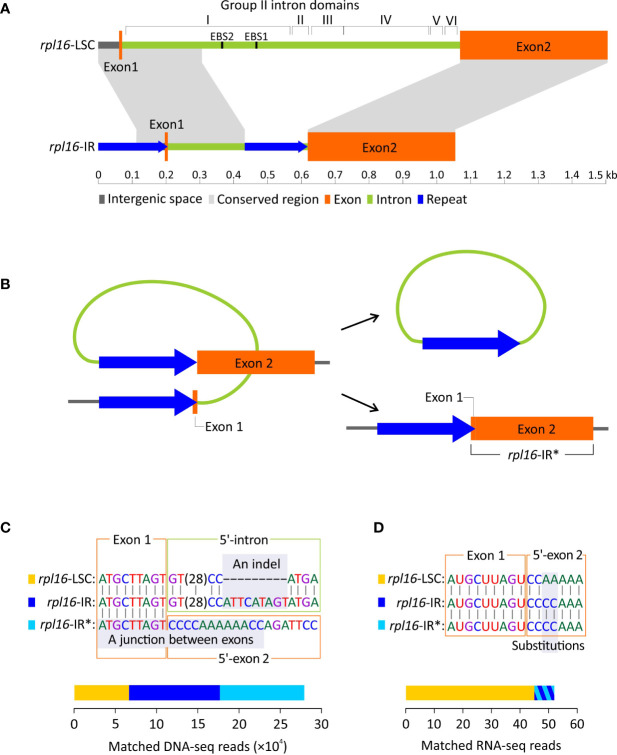
Duplication of *rpl16* in *Dichondra*. **(A)** Comparisons of *rpl16* copies residing in the LSC (*rpl16*-LSC) and IR (*rpl16*-IR) regions. The *rpl16*-LSC harbors a typical group II intron containing I−VI domains, while the *rpl16*-IR is short and degraded with only partial sequences of the domain I **(B)** A hypothetical scenario for loss of the *rpl16*-IR intron *via* homologous recombination between direct repeats. The intron-lacking *rpl16*-IR is therefore designated as *rpl16*-IR*. **(C)** DNA-seq mapping analyses showing the coexistence of three *rpl16* copies (color-coded) in *Dichondra*. Regions enabling copy discrimination are highlighted with grey and indicated with the terms “An indel” and “A junction between exons”. Stacked bars are the number of reads matching specific copies. **(D)** RNA-seq reads mapped to putative transcripts of the three *rpl16* copies after splicing. The bars for *rpl16*-IR and *rpl16*-IR* are added together because they are not distinguishable by highlighted substitutions.

As mentioned above, *Dichondra* has duplicated *rpl16* in its IRs, resulting in three *rpl16* copies in our assembly. One of these copies is located at the typical locus between *rpl14* and *rps3* in the LSC (hereafter designated as *rpl16*-LSC), while the others are located between *trnN* and *ycf1* in the IRs (hereafter designated as *rpl16*-IR). The two types of the *rpl16* copies differ in intron composition but share nearly identical coding sequences (% identical sites between different copies = 99.1). The intron of *rpl16*-LSC is 966 bp long with I−VI domains forming a typical group II intron structure (**Figure 5A**). In contrast, the *rpl16*-IR intron is relatively short (429 bp long) and completely lacks domains II−VI and EBS1 and EBS2 motifs in domain I. It is noteworthy that we detected a pair of direct repeats ─ one copy (205 bp long) in the region encompassing the exon 1 and its upstream region and the other (201 bp) at the 3’-end of the intron (**Figure 5A**). Their pairwise sequence identity is 97.6%.

The above-mentioned short, domain-lacking, but repeat-containing intron prompted us to ask two questions: (1) Is the intron in *rpl16*-IR still actively splicing? Specifically, we want to know if formation of an open reading frame is achieved after intron splicing. (2) Does homologous recombination between repeats occur and result in an intron-lacking *rpl16*-IR copy in *Dichondra* (**Figure 5B**)? The intron-lacking copy (hereafter designated as *rpl16*-IR*) is separated from other two genomic *rpl16* copies by the junction of two exons (**Figure 5C**). Furthermore, the *rpl16*-LSC and *rpl16*-IR copies are distinguishable from each other by a 9 bp long indel at the 5’-end of their introns (**Figure 5C**). Our mapping analyses reveal 6,665, 10,228, and 11,032 DNA-seq reads that match *rpl16*-LSC, *rpl16*-IR, and *rpl16*-IR*, respectively (**Figure 5C**; **Figure S3**). These results confirm that (1) at least three different *rpl16* copies coexist in the *Dichondra* plastomes, (2) the genomic copy numbers of *rpl16*-IR and *rpl16*-IR* are approximately equal but two-fold higher than *rpl16*-LSC, consistent with the fact that the former two are in the IR, while the latter is single in the LSC, and (3) the equal numbers of matched reads between *rpl16-*IR and *rpl16*-IR* copies suggest effective repeat-mediated recombination. The coexistence and copy number divergence were further verified by semi-quantitative PCR that shows specific duplicate amplicons appearing earlier than the single-copy locus (**Figure S3**).

To examine splicing capability, RNA-seq reads were mapped to putative mature transcripts of the three *rpl16* copies (**Figure 5D**). We then detected 45 RNA reads that not only spanned exon junctions but also contained substitutions specific to *rpl16*-LSC. Eight RNA reads were found to match both *rpl16*-IR and *rpl16*-IR* because the mature transcripts of these two copies are identical, leading to difficulty in identification of which copies contributed to the matched reads. Therefore, it remains uncertain whether the *rpl16*-IR’s intron is spliced. If it is, the splicing efficiency should be much lower than that in *rpl16*-LSC.

## Discussion

To date, the genus *Ipomoea* is significantly overrepresented among sequenced morning glory plastomes. This bias holds back advances in understanding the evolution and utility of plastomes across the family. Our study compared plastomes representing nine of the 12 tribes, and provides new insights into phylogeny, plastome structural variation, PTMTs, and mechanisms underlying intron loss in the family.

Monophyletic status of the family, as well as phylogenetic placements of several of its tribes and genera, are still subject to debate. We adopted the classification system modified by Stefanovi´c et al. (2003), who recognized twelve tribes within Convolvulaceae rather than nine in Austin’s system (1998). Notably, Austin (1998) raised the parasitic genus *Cuscuta* to the status of a monogeneric family (Cuscutaceae), but Stefanovi´c et al. (2003) considered it a monogeneric tribe (Cuscuteae). The present phylogenetic analyses reveal that *Cuscuta* is nested within Convolvulaceae, in line with the viewpoint that Convolvulaceae, including *Cuscuta*, is monophyletic (Stefanović et al., 2002; Stefanović and Olmstead, 2004; Lin et al., 2022; Simões et al., 2022). This monophyly is further reinforced by a common absence of the *rpl2* intron unique to Convolvulaceae among the subclass Asterideae (Stefanović et al., 2002; Lin et al., 2022; this study). However, our trees placed *Cuscuta* as a sister to other Convolvulaceous taxa except for the two genera *Dinetus* and *Erycibe* (**Figure 1**). This placement has never been reported before, even in the most recent plastid phylogenomic analysis by Lin et al. (2022). As our sampled taxa are denser and broader than those (25 taxa across eight tribes) in Lin et al. (2022), we conclude that the phylogenetic position of *Cuscuta* is unsettled and that expanded taxonomic sampling will be required to resolve *Cuscuta*’s evolutionary status.

Our analyses yielded strong support for a close relationship between Ipomoeeae *s.l.* and *Merremia* (a genus in the tribe Merremieae), confirming non-monophyly of the tribe Merremieae and calling for its revision. Furthermore, both of our ML (BS = 100%) and BI (PP = 1) trees resolve the clade comprising four genera: *Dichondra*, *Jacquemontia*, *Cressa*, and *Evolvulus*. Taxa in this clade, except *Jacquemontia*, possess deeply divided styles, the so-called “bifid style” clade or the subfamily Dicranostyloideae (Stefanović et al., 2002). Recently, the placement of *Jacquemontia* within Dicranostyloideae was recovered with full support in coalescent trees based on multiple nuclear loci (Simões et al., 2022). As a result, the placement of *Jacquemontia* within Dicranostyloideae is confirmed by a variety of methods and molecular data sets. However, the position of the genus *Erycibe* appears to be discordant among plastid, mitochondrial, and nuclear trees. For example, a sisterhood between *Dinetus* and *Erycibe* is weakly supported (BS=69%; PP = 0.994) in our trees (**Figure 1**), but such relationship was not observed in the trees inferred from mitochondrial CDSs and nuclear 45S (Lin et al., 2022). Simões et al. (2022) noted shifts of the *Erycibe* position with and without incorporation of *Cuscuta* in their analytical dataset, implicating the influence of taxonomic sampling. Collectively, our results affirm some but not all relationships in Convolvulaceae. The unresolved (or weakly supported) relationships are likely due to highly variable rates in nucleotide substitutions among taxa as demonstrated in **Figure 2**.

We identified numerous plastomic rearrangements in the LSC and IR regions (**Figure 3**). However, more than half (7/13 = 0.54) of the LSC rearrangements occurred in the subfamily Dicranostyloideae, indicating an asymmetric distribution of plastomic rearrangements across Convolvulaceae. Taxa in Dicranostyloideae also exhibit variable IRs and accelerated nucleotide substitution rates (**Figure 2**), implying an association between plastomic rearrangements and nucleotide substitution rates. This association likely results from aberrant mutation rates and/or improper DNA repair systems proposed for some Geraniaceae lineages whose plastomic rearrangements and nucleotide substitution rates are co-elevated (Guisinger et al., 2011; Blazier et al., 2016). IRs might act to stabilize plastomes (Palmer and Thompson, 1982). This hypothetical function should be ineffective in Convolvulaceae. Blazier et al. (2016) suggested that plastome stabilization by IRs is evident when repeat content is low. Indeed, we have detected a pair of direct repeats and shown its capability of efficiently triggering recombination (**Figure 5**). We propose that multiple independent rounds of IR contraction and expansion have shaped the evolution of gene content in the IRs of Convolvulaceae. Illegitimate recombination between IR and single copy regions might have led to IR contraction and expansion (Goulding et al., 1996). Although not involved in gene losses, IR size fluctuation has resulted in copy-number variation of some genes in some Convolvulaceae plastomes (**Figure 3**). The low expression level we observed in *rpl16*-IR and *rpl16*-IR* implies down-regulation of these duplicates (**Figure 5**). Whether expression regulation also acts on other duplicate IR resident loci to prevent (or mediate) dosage effects remains to be elucidated.

In this study we report the first PTMT case in Convolvulaceae (**Figure 4**). This PTMT replaces the plastid intergenic sequences between *psaA* and *ycf3* in *Jacquemontia*. Replacements of plastid sequences with PTMTs were previously documented in *Daucus* (Iorizzo et al., 2012a; Iorizzo et al., 2012b), *Pariana* (Ma et al., 2015), and *Convallaria* (Raman et al., 2021). In *Jacquemontia*, the PTMT is inserted upstream of the *psaA* operon consisting of *psaA*, *psaB*, and *rps14* (Meng et al., 1988). This insertion has interrupted the transcription of the *psaA* operon driven by the original plastid promoters (**Figure 5**). Therefore, the expression machinery of the *psaA* operon must be reshaped after the PTMT insertion to ensure regular transcription of *psaA* and *psaB*, for their products are essential for the assembly of the PSI complex. In *Daucus*, replacements of plastid promoters with PTMTs were hypothesized to alter the expression pattern of the corresponding genes (Iorizzo et al., 2012a). A recent study suggested that mis-regulation of the plastid *psbB* operon results in hybrid incompatibility that may ultimately lead to speciation in *Oenothera* (Zupok et al., 2021). The PTMT we identified in *Jacquemontia* provides an opportunity to assess the association between PTMT insertions and speciation.

A variety of mechanisms can underlie PTMT insertions. The presence of short repeats and transcriptase-like sequences leads to the conclusion that the PTMT in *Daucus* was a consequence of transposition events governed by non-LTR retrotransposons (Iorizzo et al., 2012a; Iorizzo et al., 2012b). In *Asclepias*, migrations of mtDNA to plastids were achieved by homologous recombination between mitochondrial plastid-derived DNA and plastid sequences (Straub et al., 2013). Previously, deletions adjacent to insertion sites were considered to be signatures of homologous recombination facilitating PTMT integration in *Anacardium* (Rabah et al., 2017). Nevertheless, neither transposable elements nor deletions were detected in the PTMT or its flanking regions in *Jacquemontia*. Surprisingly, our blast searches revealed that the *Jacquemontia* PTMT contains several regions that best match mitogenomes of diverse species across five taxonomic orders (Amborellales, Fabales, Lamiales, Malpighiales, and Solanales; **Figure 4**). It is implausible to assume multiple rounds of horizontal gene transfer (HGT) from different origins into the same plastid intergenic region. Several studies have shown that PTMTs preserve the ancestral state of their mitochondrial donors (e.g., Iorizzo et al., 2012a; Iorizzo et al., 2012b; Straub et al., 2013; Ma et al., 2015). Unfortunately, we did not obtain any matches when the PTMT of *Jacquemontia* was used to blast against the mtDNA scaffolds we generated from conspecific PE reads. It is known that land plant mitogenomes can acquire abundant foreign DNA from intercellular gene transfer (IGT), HGT, or both (Knoop, 2004; Alverson et al., 2010; Rice et al., 2013; Park et al., 2014). Accordingly, we propose a straightforward scenario that the *Jacquemontia* PTMT has retained the ancestral state of its mitochondrial donor that once harbored an array of foreign DNA of different origins but later experienced reduction by purging the sequences homologous to this PTMT.

We found a short group II intron in the *rpl16*-IR copy (**Figure 5**). This intron is degraded and likely not functional since it lacks most of the domains, including the domain V for binding catalytic Mg^2+^ during intron splicing (Lambowitz and Zimmerly, 2011). This degraded intron is flanked by direct repeats capable of triggering homologous recombination to precisely remove it. This repeat-mediated recombination happens with a rate of 0.52 [11,032/(10,228 + 11,032)] conversions per copy and its associated product is transcribable, albeit at a low level. These findings lead us to speculate that these direct repeats might have been selected to complement the degraded intron since the intron is nonfunctional.

Retroprocessing is a well-known mechanism driving intron loss in plant organelles through conversion between retro-transcribed cDNA molecules and their corresponding DNA fragments. This mechanism is especially prominent when transcripts of target genes lack RNA editing in regions adjacent to former splicing sites (Ran et al., 2010; Sloan et al., 2010; Grewe et al., 2011; Cuenca et al., 2016). In addition, HGT and sequential gene conversion have created a chimeric *cox2* gene without introns (Hepburn et al., 2012). Here, we provide a different model for intron loss after invasion of direct repeats at appropriate positions in intron-containing genes. This model better interprets intron loss from non-chimeric genes that have abundant RNA editing sites in regions adjacent to the former intron splicing sites.

## Data availability statement

The original contributions presented in the study are publicly available. This data can be found here: NCBI, LC729542-LC729560 and the SRA bioproject: PRJNA876072.

## Author contributions

S-MC and C-SW conceived and designed the study. C-SW performed experiments and data analyses. C-SW and C-IC collected the plant materials. C-SW and S-MC wrote the manuscript. All the authors checked and approved the final version. All authors contributed to the article and approved the submitted version.

## Supplementary material

Supplementary Figure 1 Plastid phylogenomics of the tribe Ipomoeeae *s.l.* The tree details the simplified area depicted in . A 50% majority rule was used to condense the tree topology. Values along branches are bootstrap supports (%) and posterior possibilities for ML and Bayesian inference (BI) trees, respectively. Conflicting topologies are highlighted with grey.

Supplementary Figure 2 Dating of taxon divergence time during Convolvulaceae evolution. Black circles indicate nodes with constrained ages. Blue bars denote a range of the 95% highest posterior density (HPD).

Supplementary Figure 3 Read-mapping and Semi-quantitative PCR demonstrating coexistence of the three different *rpl16* copies and discrepancies in their copy numbers in *Dichondra*. Primers, *rpl16*-LSC-F and *rpl16*-LSC-R, were designed to amplify the region containing intronic II−IV domains that are lacking in the *rpl16*-IR copy.
