# Supplementary figures and images for: Plastid phylogenomics and plastome evolution in the morning glory family (Convolvulaceae)
Source: Front Plant Sci. 2022 Dec 20;13:1061174. doi: 10.3389/fpls.2022.1061174 (PMC9808526; doi:10.3389/fpls.2022.1061174)

Figure S1

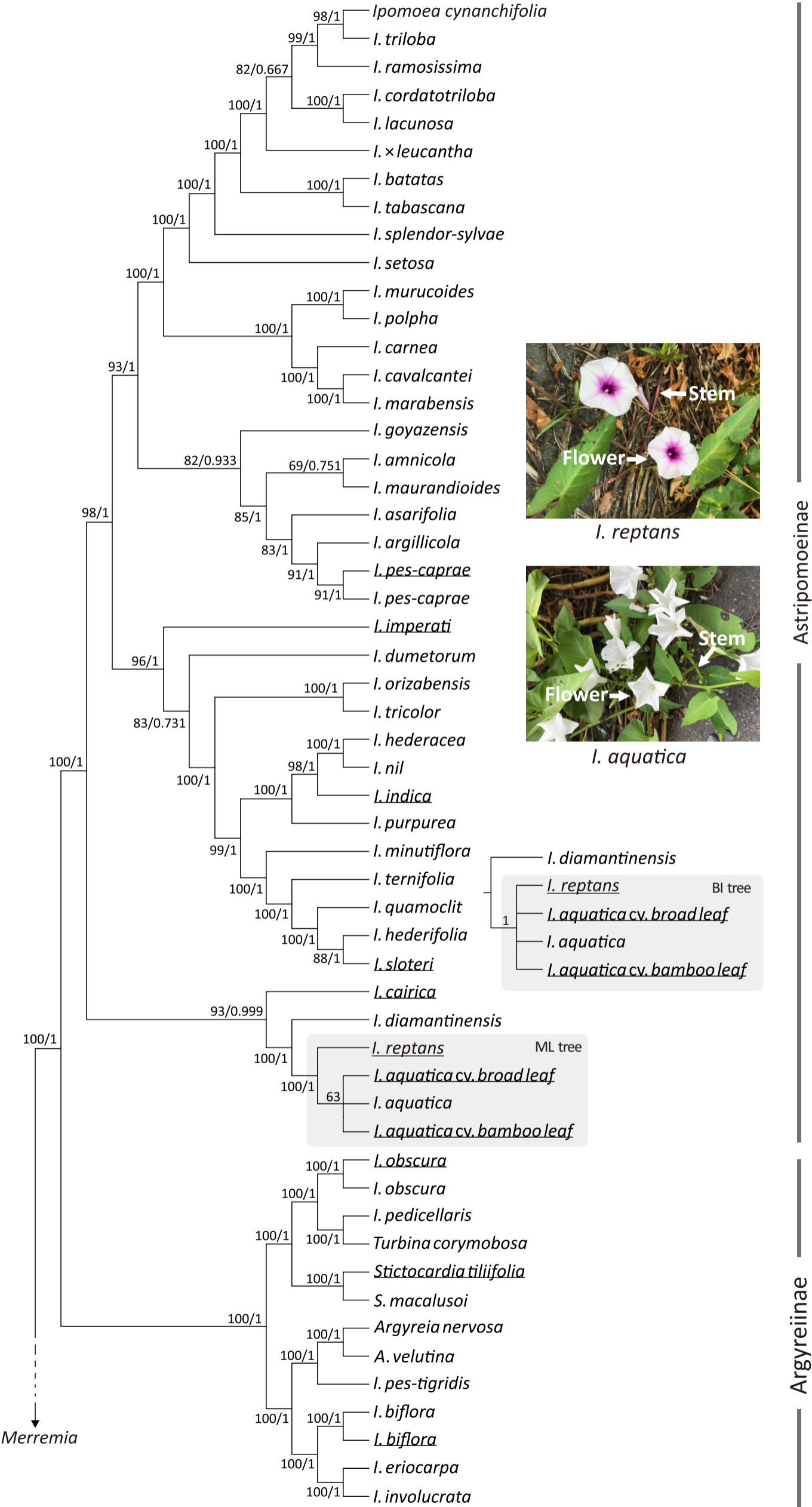

Supplement: Supplementary Figure 1 — Plastid phylogenomics of the tribe Ipomoeeae s.l. The tree details the simplified area depicted in . A 50% majority rule was used to condense the tree topology. Values along branches are bootstrap supports (%) and posterior possibilities for ML and Bayesian inference (BI) trees, respectively. Conflicting topologies are highlighted with grey. [file DataSheet_1.pdf]

Figure S2

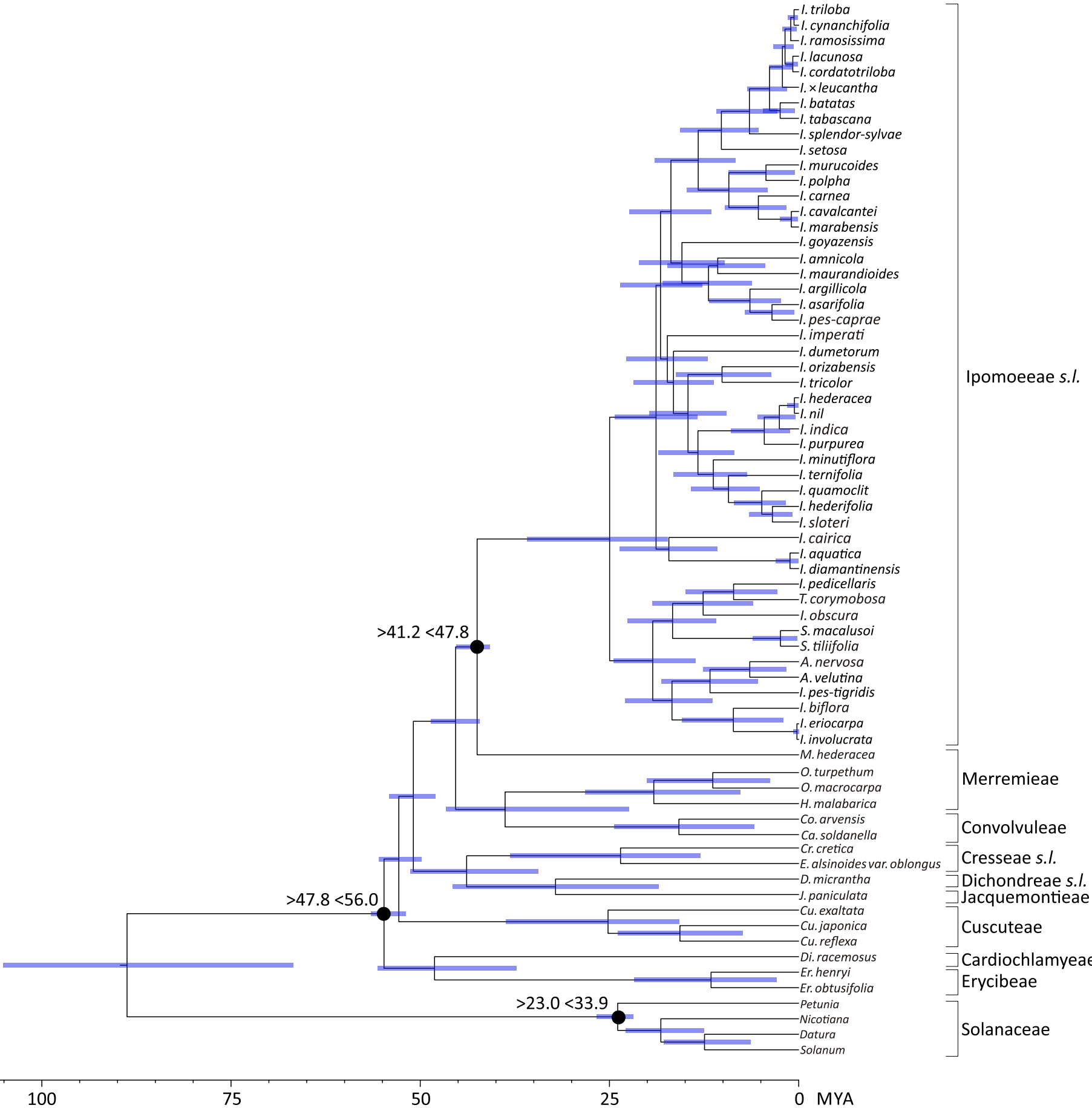

Supplement: Supplementary Figure 2 — Dating of taxon divergence time during Convolvulaceae evolution. Black circles indicate nodes with constrained ages. Blue bars denote a range of the 95% highest posterior density (HPD). [file DataSheet_2.pdf]

Figure S3

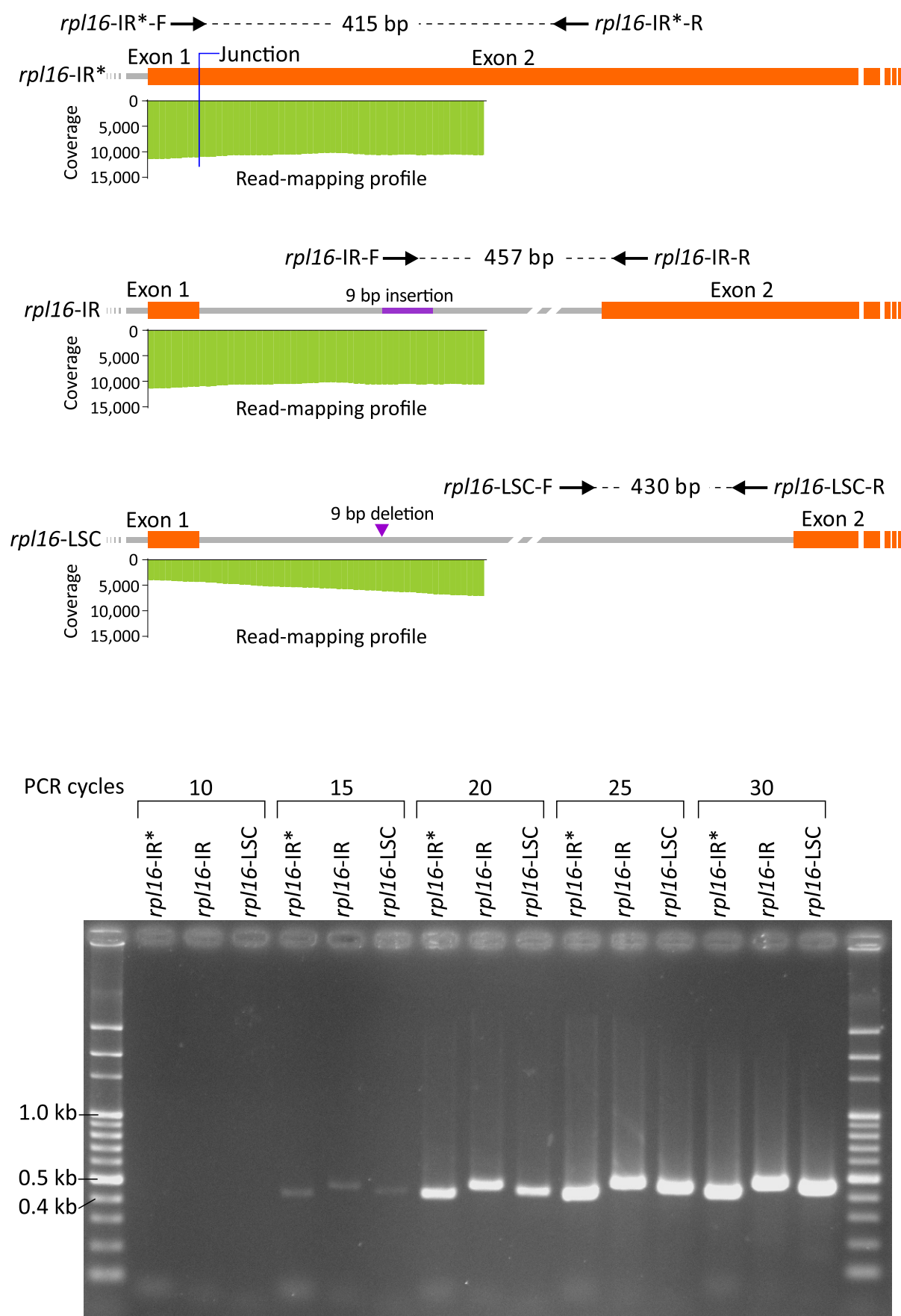

Supplement: Supplementary Figure 3 — Read-mapping and Semi-quantitative PCR demonstrating coexistence of the three different rpl16 copies and discrepancies in their copy numbers in Dichondra. Primers, rpl16-LSC-F and rpl16-LSC-R, were designed to amplify the region containing intronic II−IV domains that are lacking in the rpl16-IR copy. [file DataSheet_3.pdf]
