# Supplementary material for: Plastid phylogenomics and plastome evolution in the morning glory family (Convolvulaceae)
Source: Front Plant Sci. 2022 Dec 20;13:1061174. doi: 10.3389/fpls.2022.1061174 (PMC9808526; doi:10.3389/fpls.2022.1061174)
Supplement: Supplementary file 4 [file Table_1.docx]

| Table S1. GPS information and voucher numbers of the sequenced taxa | | |
| --- | --- | --- |
| Species | GPS information | Voucher number |
| *Calystegia soldanella* | 25.24216440975708, 121.63382282171845 | Chaw 1587 |
| *Dichondra micrantha* | 25.110432695161162, 121.92084352801459 | Chaw 1585 |
| *Erycibe henryi* | 24.214963430467378, 120.73835181560715 | Chaw 1592 |
| *Evolvulus alsinoides var. oblongus* | 25.095047461482455, 121.91749363707486 | Chaw 1594 |
| *Hewittia malabarica* | 21.956064334275652, 120.82001014484972 | Chaw 1595 |
| *Ipomoea aquatica cv. bamboo leaf* | 22.429228610691716, 120.51411639580317 | Chaw 1577 |
| *Ipomoea aquatica cv. broad leaf* | 22.44583275634831, 120.50942687279823 | Chaw 1604 |
| *Ipomoea biflora* | 24.458608392546996, 118.39518638295381 | Chaw 1599 |
| *Ipomoea cairica* | 25.04196829967557, 121.61302246486586 | Chaw 1603 |
| *Ipomoea imperati* | 23.08203180698311, 120.03714632761738 | Chaw 1576 |
| *Ipomoea indica* | 22.4298548949015, 120.48817723815223 | Chaw 1574 |
| *Ipomoea obscura* | 22.446409220536204, 120.52243069033102 | Chaw 1579 |
| *Ipomoea pes-caprae* | 22.417859695511815, 120.50594694416056 | Chaw 1583 |
| *Ipomoea reptans* | 22.459735667649543, 120.53882650846484 | Chaw 1578 |
| *Ipomoea sloteri* | 25.04280771702921, 121.61123437143188 | Chaw 1573 |
| *Jacquemontia paniculata* | 22.417116053973274, 120.66844632940612 | Chaw 1598 |
| *Merremia hederacea* | 22.450439152140568, 120.53084343559995 | Chaw 1582 |
| *Operculina turpethum* | 22.423998514589154, 120.51425036100267 | Chaw 1581 |
| *Stictocardia tiliifolia* | 22.94443482001025, 120.63428131282272 | Chaw 1593 |
