# Supplementary material for: Plastid phylogenomics and plastome evolution in the morning glory family (Convolvulaceae)
Source: Front Plant Sci. 2022 Dec 20;13:1061174. doi: 10.3389/fpls.2022.1061174 (PMC9808526; doi:10.3389/fpls.2022.1061174)
Supplement: Supplementary file 5 [file Table_2.docx]

| Table S2. Plastoimc data used in this study | | | | | |
| --- | --- | --- | --- | --- | --- |
| Family | Species | Assembly status | Size | GC content | GenBank accession |
| Convolvulaceae | *Argyreia nervosa* | Partial genome | 159,847 | 37.60% | KF242477 |
|  | *Argyreia velutina* | Complete genome | 162,848 | 37.50% | NC060787 |
|  | *Calystegia soldanella* | Complete genome | 152,317 | 37.80% | NC060788 |
|  | *Calystegia soldanella** | Complete genome | 152,365 | 37.70% | LC729542 |
|  | *Convolvulus arvensis* | Complete genome | 153,234 | 37.70% | NC054224 |
|  | *Convolvulus arvensis* | Complete genome | 153,234 | 37.70% | NC054224 |
|  | *Cressa cretica* | Complete genome | 141,419 | 38.60% | NC035516 |
|  | *Cuscuta exaltata* | Complete genome | 125,373 | 38.10% | NC009963 |
|  | *Cuscuta japonica* | Complete genome | 121,037 | 38.30% | MH780080 |
|  | *Cuscuta japonica* | Complete genome | 120,975 | 38.30% | MZ240742 |
|  | *Cuscuta reflexa* | Complete genome | 121,521 | 38.20% | NC009766 |
|  | *Dichondra micrantha* | Complete genome | 163,228 | 37.70% | NC060790 |
|  | *Dichondra micrantha** | Complete genome | 162,315 | 37.70% | LC729543 |
|  | *Dinetus racemosus* | Complete genome | 150,706 | 39.00% | NC060791 |
|  | *Erycibe henryi** | Complete genome | 155,683 | 37.70% | LC729544 |
|  | *Erycibe obtusifolia* | Complete genome | 150,333 | 37.90% | NC060792 |
|  | *Evolvulus alsinoides* | Complete genome | 157,015 | 37.40% | NC058590 |
|  | *Evolvulus alsinoides* | Complete genome | 157,015 | 37.40% | NC058590 |
|  | *Evolvulus alsinoides var. oblongus** | Complete genome | 162,367 | 37.30% | LC729545 |
|  | *Hewittia malabarica** | Complete genome | 153,219 | 37.70% | LC729546 |
|  | *Ipomoea amnicola* | Partial genome | 161,172 | 37.60% | KF242478 |
|  | *Ipomoea aquatica* | Complete genome | 162,663 | 37.40% | NC056300 |
|  | *Ipomoea aquatica cv. bamboo leaf** | Complete genome | 162,390 | 37.50% | LC729548 |
|  | *Ipomoea aquatica cv. broad leaf** | Complete genome | 161,780 | 37.60% | LC729549 |
|  | *Ipomoea argillicola* | Partial genome | 161,119 | 37.60% | KF242479 |
|  | *Ipomoea asarifolia* | Complete genome | 160,589 | 37.50% | NC042935 |
|  | *Ipomoea batatas* | Complete genome | 161,303 | 37.60% | NC026703 |
|  | *Ipomoea biflora* | Complete genome | 153,203 | 37.70% | NC060786 |
|  | *Ipomoea biflora** | Complete genome | 153,167 | 37.70% | LC729550 |
|  | *Ipomoea cairica** | Complete genome | 161,780 | 37.60% | LC729551 |
|  | *Ipomoea carnea* | Complete genome | 160,819 | 37.60% | NC042936 |
|  | *Ipomoea cavalcantei* | Complete genome | 161,563 | 37.60% | NC042937 |
|  | *Ipomoea cordatotriloba* | Complete genome | 161,242 | 37.60% | NC041204 |
|  | *Ipomoea cynanchifolia* | Complete genome | 161,386 | 37.50% | NC041203 |
|  | *Ipomoea diamantinensis* | Partial genome | 161,266 | 37.50% | KF242481 |
|  | *Ipomoea dumetorum* | Partial genome | 161,191 | 37.60% | KF242482 |
|  | *Ipomoea eriocarpa* | Partial genome | 160,976 | 37.50% | KF242483 |
|  | *Ipomoea goyazensis* | Complete genome | 160,414 | 37.60% | NC042938 |
|  | *Ipomoea hederacea* | Complete genome | 161,354 | 37.50% | NC037911 |
|  | *Ipomoea hederifolia* | Partial genome | 161,383 | 37.50% | KF242484 |
|  | *Ipomoea imperati** | Complete genome | 161,861 | 37.50% | LC729552 |
|  | *Ipomoea indica** | Complete genome | 162,077 | 37.50% | LC729553 |
|  | *Ipomoea involucrata* | Partial genome | 160,531 | 37.50% | KF242485 |
|  | *Ipomoea lacunosa* | Complete genome | 161,492 | 37.50% | NC037912 |
|  | *Ipomoea marabensis* | Complete genome | 161,324 | 37.50% | NC042939 |
|  | *Ipomoea maurandioides* | Complete genome | 161,242 | 37.50% | NC042940 |
|  | *Ipomoea minutiflora* | Partial genome | 161,190 | 37.60% | KF242498 |
|  | *Ipomoea murucoides* | Partial genome | 160,072 | 37.60% | KF242486 |
|  | *Ipomoea nil* | Complete sequence | 161,897 | 37.50% | NC031159 |
|  | *Ipomoea obscura* | Partial genome | 161,250 | 37.50% | KF242499 |
|  | *Ipomoea obscura** | Complete genome | 159,578 | 37.50% | LC729554 |
|  | *Ipomoea orizabensis* | Partial genome | 160,940 | 37.60% | KF242488 |
|  | *Ipomoea pedicellaris* | Partial genome | 160,657 | 37.60% | KF242489 |
|  | *Ipomoea pes-caprae* | Partial genome | 160,168 | 37.60% | KF242490 |
|  | *Ipomoea pes-caprae** | Complete genome | 161,618 | 37.60% | LC729555 |
|  | *Ipomoea pes-tigridis* | Partial genome | 162,140 | 37.60% | KF242500 |
|  | *Ipomoea polpha* | Partial genome | 161,205 | 37.50% | KF242491 |
|  | *Ipomoea purpurea* | Complete genome | 162,046 | 37.50% | NC009808 |
|  | *Ipomoea quamoclit* | Complete genome | 160,836 | 37.50% | NC042941 |
|  | *Ipomoea ramosissima* | Complete genome | 161,402 | 37.50% | NC041205 |
|  | *Ipomoea reptans** | Complete genome | 162,730 | 37.40% | LC729547 |
|  | *Ipomoea setosa* | Partial genome | 161,035 | 37.60% | KF242492 |
|  | *Ipomoea sloteri** | Complete genome | 161,841 | 37.50% | LC729557 |
|  | *Ipomoea splendor-sylvae* | Complete genome | 161,721 | 37.50% | NC041206 |
|  | *Ipomoea tabascana* | Complete genome | 161,225 | 37.60% | NC041207 |
|  | *Ipomoea ternifolia* | Partial genome | 161,409 | 37.50% | KF242494 |
|  | *Ipomoea tricolor* | Partial genome | 160,776 | 37.60% | KF242495 |
|  | *Ipomoea triloba* | Complete genome | 161,750 | 37.50% | NC037913 |
|  | *Ipomoea × leucantha* | Complete genome | 161,296 | 37.50% | NC041208 |
|  | *Jacquemontia paniculata** | Complete genome | 164,697 | 38.80% | LC729558 |
|  | *Merremia hederacea* | Complete genome | 155,308 | 37.60% | NC060793 |
|  | *Merremia hederacea** | Complete genome | 154,711 | 37.70% | LC729559 |
|  | *Operculina macrocarpa* | Partial genome | 161,475 | 37.60% | KF242502 |
|  | *Operculina turpethum** | Complete genome | 153,970 | 37.80% | LC729560 |
|  | *Stictocardia macalusoi* | Complete genome | 162,849 | 37.50% | KF242503 |
|  | *Stictocardia tiliifolia** | Complete genome | 165,459 | 37.40% | LC729556 |
|  | *Turbina corymbosa* | Complete genome | 161,102 | 37.70% | KF242504 |
| Solanaceae | *Datura stramonium* | Complete genome | 155,871 | 37.90% | NC018117 |
|  | *Nicotiana suaveolens* | Complete genome | 155,883 | 37.80% | NC056978 |
|  | *Petunia exserta* | Complete genome | 156,597 | 37.80% | NC050782 |
|  | *Solanum limbaniense* | Complete genome | 155,508 | 37.90% | NC041617 |
| *: Sequenced in this study | | | | | |
