# Supplementary material for: Plastid phylogenomics and plastome evolution in the morning glory family (Convolvulaceae)
Source: Front Plant Sci. 2022 Dec 20;13:1061174. doi: 10.3389/fpls.2022.1061174 (PMC9808526; doi:10.3389/fpls.2022.1061174)
Supplement: Supplementary file 6 [file Table_3.docx]

| Table S3. Estimated values of absolute synonymous (*RS*) and nonsynonymous (*RN*) substitution rates | | | |
| --- | --- | --- | --- |
| Tribe/genus | Species | RS (Substitutions per codon per billion year) | RN (Substitutions per codon per billion year) |
| Ipomoeeae *s.l.* | *Ipomoea triloba* | 0.017636 | 0.004899 |
|  | *Ipomoea cynanchifolia* | 0.070705 | 0 |
|  | *Ipomoea ramosissima* | 0.020628 | 0.005826 |
|  | *Ipomoea lacunosa* | 0.041968 | 0.003874 |
|  | *Ipomoea cordatotriloba* | 0.027893 | 0.003874 |
|  | *Ipomoea × leucantha* | 0.055398 | 0.002837 |
|  | *Ipomoea batatas* | 0.035371 | 0.033452 |
|  | *Ipomoea tabascana* | 0.013275 | 0.004493 |
|  | *Ipomoea splendor-sylvae* | 0.02165 | 0.005147 |
|  | *Ipomoea setosa* | 0.029742 | 0.004846 |
|  | *Ipomoea murucoides* | 0.030065 | 0.000694 |
|  | *Ipomoea polpha* | 0.022543 | 0.003518 |
|  | *Ipomoea carnea* | 0.03457 | 0.005893 |
|  | *Ipomoea cavalcantei* | 0.000103 | 0.003083 |
|  | *Ipomoea marabensis* | 0.0111 | 0.025077 |
|  | *Ipomoea goyazensis* | 0.021801 | 0.010479 |
|  | *Ipomoea amnicola* | 0.017223 | 0.002562 |
|  | *Ipomoea maurandioides* | 0.010126 | 0.007125 |
|  | *Ipomoea argillicola* | 0.002329 | 0 |
|  | *Ipomoea asarifolia* | 0.078406 | 0.040578 |
|  | *Ipomoea pes-caprae* | 0.01364 | 0.00811 |
|  | *Ipomoea imperati* | 0.024156 | 0.005791 |
|  | *Ipomoea dumetorum* | 0.042293 | 0.006951 |
|  | *Ipomoea orizabensis* | 0.039709 | 0.006705 |
|  | *Ipomoea tricolor* | 0.035394 | 0.008039 |
|  | *Ipomoea hederacea* | 0.095634 | 0 |
|  | *Ipomoea nil* | 0.001061 | 0 |
|  | *Ipomoea indica* | 0.021192 | 0.005954 |
|  | *Ipomoea purpurea* | 0.049959 | 0.00803 |
|  | *Ipomoea minutiflora* | 0.055935 | 0.008112 |
|  | *Ipomoea ternifolia* | 0.081037 | 0.014969 |
|  | *Ipomoea quamoclit* | 0.025458 | 0.01585 |
|  | *Ipomoea hederifolia* | 0.036088 | 0.009792 |
|  | *Ipomoea sloteri* | 0.029435 | 0.013018 |
|  | *Ipomoea cairica* | 0.020271 | 0.004979 |
|  | *Ipomoea aquatica* | 0.04732 | 0 |
|  | *Ipomoea diamantinensis* | 0.009237 | 0.005316 |
|  | *Ipomoea pedicellaris* | 0.034099 | 0.008859 |
|  | *Turbina corymbosa* | 0.06192 | 0.012765 |
|  | *Ipomoea obscura* | 0.03612 | 0.006986 |
|  | *Stictocardia macalusoi* | 0.004444 | 0.001246 |
|  | *Stictocardia tiliifolia* | 0.032269 | 0.023838 |
|  | *Argyreia nervosa* | 0.029416 | 0.012921 |
|  | *Argyreia velutina* | 0.029323 | 0.013029 |
|  | *Ipomoea pes-tigridis* | 0.033422 | 0.004643 |
|  | *Ipomoea biflora* | 0.021811 | 0.004126 |
|  | *Ipomoea eriocarpa* | 0.002621 | 0 |
|  | *Ipomoea involucrata* | 0.045435 | 0 |
| Merremieae | *Merremia hederacea* | 0.022835 | 0.00471 |
|  | *Hewittia malabarica* | 0.045084 | 0.011775 |
|  | *Operculina macrocarpa* | 0.044681 | 0.010346 |
|  | *Operculina turpethum* | 0.040151 | 0.008883 |
| Convolvuleae | *Calystegia soldanella* | 0.054773 | 0.0091 |
|  | *Convolvulus arvensis* | 0.064544 | 0.011325 |
| Dichondreae *s.l.* | *Dichondra micrantha* | 0.185678 | 0.053124 |
| *Jacquemontia* | *Jacquemontia paniculata* | 0.179819 | 0.064539 |
| Cresseae *s.l.* | *Cressa cretica* | 0.263333 | 0.064722 |
|  | *Evolvulus alsinoides* var. *oblongus* | 0.200973 | 0.060551 |
| *Cuscuta* | *Cuscuta exaltata* | 0.095062 | 0.026217 |
|  | *Cuscuta japonica* | 0.130755 | 0.036689 |
|  | *Cuscuta reflexa* | 0.156189 | 0.053476 |
| *Dinetus* | *Dinetus racemosus* | 0.149482 | 0.041156 |
| *Erycibe* | *Erycibe henryi* | 0.02459 | 0.01768 |
|  | *Erycibe obtusifolia* | 0.019701 | 0.006797 |
